# Production of Vitamin D_3_ Enriched Biomass of *Saccharomyces Cerevisiae* as A Potential Food Supplement: Evaluation and Optimization of Culture Conditions Using Plackett–Burman and Response Surface Methodological Approaches

**DOI:** 10.22037/ijpr.2019.1100660

**Published:** 2019

**Authors:** Morteza Mohajeri Amiri, Mohammad Reza Fazeli, Tahereh Babaee, Mohsen Amini, Nasim Hayati Roodbari, Seyed Babak Mousavi, Nasrin Samadi

**Affiliations:** a *Department of Biology, Science and Research Branch, Islamic Azad University, Tehran, Iran. *; b *Department of Drug and Food Control, Faculty of Pharmacy, Tehran University of Medical Sciences, Tehran, Iran*; c *Department of Medicinal Chemistry, Faculty of Pharmacy, Tehran University of Medical Sciences, Tehran, Iran.*; d *Biotechnology Group, Chemical Engineering Department, Tarbiat Modares University, Tehran, Iran.*; e *Pharmaceutical Quality Assurance Research Center, The Institute of Pharmaceutical Sciences (TIPS), Tehran University of Medical Sciences, Tehran, Iran.*

**Keywords:** Vitamin D3, Saccharomyces cerevisiae, Food supplement, Optimization, Cholecalciferol

## Abstract

Vitamin D deficiency causes osteoporosis, osteopenia, fractures, rickets, and more recently is linked with some chronic illnesses such as cancer. Because of the safety and probiotic properties of the yeast *Saccharomyces cerevisiae*, we hypothesized that yeast cells enriched with cholecalciferol (vitamin D_3_) could represent a solution for prevention or treatment of vitamin D deficiency. In this study *S. cerevisiae* was used as a vitamin D_3 _accumulator for the first time and the optimal conditions for enrichment of* S. cerevisiae* were determined. The Plackett-Burman screening studies were used for selection of the most important factors affecting cholecalciferol entrapment. Response surface methodology was employed for optimization of cholecalciferol accumulation in *S. cerevisiae* cells by using Box-Behnken design. A modified quadratic polynomial model fit the data appropriately. The optimal points of variables to maximize the response were cholecalciferol initial concentration of 358021.16 IU/mL, tryptone concentration of 1.82 g/L, sucrose concentration of 7.13 % (w/v), and shaking speed of 140.46 rpm. The maximum amount of cholecalciferol in dry cell weight of *S. cerevisiae* was 4428.11 IU/g. The cholecalciferol entrapment in yeast biomass increased about two-folds in optimized condition which indicates efficiency of optimization.

## Introduction

Yeast enrichment refers to a method by which micronutrients and essential elements are increased in the yeast biomass (1). The baker’s yeast, *Saccharomyces cerevisiae,* is one of the popular eukaryotic microorganisms which have been recognized since ancient times (2, 3). Due to the short generation time, growing in minimal media and common environmental conditions and extraordinary fermentation properties, yeast cells are employed in the biotechnology and food industries (2, 4). *S. cerevisiae* has been introduced as a probiotic (5) for animals and human which has already indicated anti-inflammatory properties in induced colitis in mice (6) and ability in reducing digestive pain in patients with gastrointestinal disorders especially irritable bowel syndrome (IBS) (7).

Calciferol (vitamin D) is a family of fat soluble substances that increases intestinal absorption of magnesium, iron, phosphate, and zinc in vertebrates (8). Under the influence of solar UV-B radiation, this substance is synthesized from cholesterol in the dermal tissues (9). Nowadays, calciferol deficiency is distinguished as a pandemic. The main reason is inadequate sun exposure for most humans and another reason is lack of calciferol in foods (10). Calciferol deficiency causes some diseases in adults and children such as osteoporosis, osteopenia, fractures, and rickets (11). Several studies indicated that taking calciferol supplements had positive effects on pregnancy outcomes and decreased cancer mortality (12, 13).

**Table 1:** Parameters employed in screening by the Plackett-Burman design

**Parameters**	**Unit**	**Code**	**Low level (‒1)**	**High level (+1)**
Temperature	°C	A	25	40
Shaking speed	Rpm	B	100	200
Initial cholecalciferol concentration	IU/ml	C	100000	400000
Tryptone	g/l	D	0.5	2.0
Added dextrose	% w/v	E	2	10
Inoculation volume	Cfu	F	103	107
Initial pH	-	G	4.5	7.5
Sucrose	% w/v	H	2	8

**Table 2:** The Plackett-Burman screening design and responses*.

			**Coded parameters**										**Response**
**Trial number**	**A**	**B**	**C**	**D**	**E**	**F**	**G**	**H**	**J**	**K**	**L**		**Cholecalciferol in dry cell weight (IU/g)**
1	40.00	200.00	400000.00	0.5	2	10^3^	7.50	2.00	+1	-1	-1		1086.81
2	40.00	100.00	400000.00	2	2	10^3^	4.50	8.00	+1	+1	-1		1450.21
3	25.00	100.00	400000.00	0.5	10	10^7^	7.50	2.00	+1	+1	+1		1073.23
4	25.00	200.00	100000.00	2	10	10^3^	4.50	2.00	+1	-1	+1		1285.23
5	40.00	200.00	400000.00	2	10	10^7^	4.50	2.00	-1	+1	-1		1438.1
6	25.00	100.00	400000.00	2	10	10^3^	7.50	8.00	-1	-1	+1		1467.32
7	40.00	200.00	100000.00	0.5	10	10^3^	7.50	8.00	-1	+1	-1		1137.59
8	40.00	100.00	100000.00	2	2	10^7^	7.50	2.00	-1	-1	+1		1050.33
9	40.00	100.00	100000.00	0.5	10	10^7^	4.50	8.00	-1	-1	-1		837.49
10	25.00	200.00	400000.00	0.5	2	10^7^	4.50	8.00	+1	-1	-1		1203.15
11	25.00	100.00	100000.00	0.5	2	10^3^	4.50	2.00	+1	+1	+1		789.32
12	25.00	200.00	100000.00	2	2	10^7^	7.50	8.00	-1	+1	+1		1437.07
13	32.50	150.00	250000.00	1.25	6	5.0005×10^6^	6.00	5.00	0	0	0		1127.54
14	32.50	150.00	250000.00	1.25	6	5.0005×10^6^	6.00	5.00	0	0	0		1149.53
15	32.50	150.00	250000.00	1.25	6	5.0005×10^6^	6.00	5.00	0	0	0		1129.17
16	32.50	150.00	250000.00	1.25	6	5.0005×10^6^	6.00	5.00	0	0	0		1132.34
17	32.50	150.00	250000.00	1.25	6	5.0005×10^6^	6.00	5.00	0	0	0		1123.02

* Coded parameters (A to H) were described in text. J, K and L codes are dummy parameters. Five last experiments are in central points.

**Table 3:** The most significant parameters used in Box-Behnken experimental design

Parameters	Unit	Code	Low level (‒1)	Basal level (0)	High level (+1)
Initial cholecalciferol concentration	IU/ml	A	100000	250000	400000
Tryptone	g/l	B	0.5	1.25	2.0
Sucrose	%w/v	C	2.0	5.0	8.0
Shaking speed	rpm	D	100	150	200

**Table 4:** Coefficient estimation of selected parameters on cholecalciferol uptake in dry cell weight of *S. cerevisiae *employing the Plackett-Burman design

**Parameters**	**Symbol**	**Coefficient estimate**	**F-value**	***P*** **-value Prob >F**
Temperature	A	-11.74	9.80	0.0259
Shaking speed	B	76.67	627.08	< 0.0001
Initial cholecalciferol concentration	C	88.99	563.16	< 0.0001
Tryptone	D	176.22	2208.32	< 0.0001
Added dextrose	E	28.00	55.76	0.0007
Inoculation volume	F	-5.27	1.97	0.2193
pH	G	30.23	65.00	0.0005
Sucrose	H	76.98	421.41	< 0.0001

**Table 5:** The Box-Behnken design and results of experiments.

		**Parameters**			**Response**
**Run**	**A: Initial cholecalciferol** **concentration (IU/ml)**	**B: Tryptone (g/l)**	**C: Sucrose** **% (w/v)**	**D: Shaking** **speed (rpm)**	**Cholecalciferol per** **dry cell weight (IU/g)**
1	250000.00	2.00	5.00	100.00	2629.61
2	250000.00	0.50	5.00	100.00	1321.96
3	250000.00	1.25	2.00	100.00	1144.07
4	100000.00	1.25	2.00	150.00	1566.53
5	250000.00	0.50	2.00	150.00	1228.04
6	250000.00	1.25	2.00	200.00	1405.95
7	250000.00	0.50	5.00	200.00	856.59
8	100000.00	0.50	5.00	150.00	1182.94
9	100000.00	2.00	5.00	150.00	1825.58
10	100000.00	1.25	5.00	100.00	1325.95
11	250000.00	1.25	5.00	150.00	3675.29
12	250000.00	1.25	5.00	150.00	3633.74
13	250000.00	1.25	5.00	150.00	3806.85
14	100000.00	1.25	8.00	150.00	1917.69
15	250000.00	2.00	5.00	200.00	1967.35
16	250000.00	0.50	8.00	150.00	1649.03
17	100000.00	1.25	5.00	200.00	785.331
18	400000.00	2.00	5.00	150.00	3880.96
19	400000.00	1.25	2.00	150.00	2263.93
20	250000.00	1.25	5.00	150.00	3629.65
21	400000.00	1.25	5.00	100.00	2380.49
22	250000.00	2.00	8.00	150.00	3753.4
23	400000.00	0.50	5.00	150.00	1775.68
24	400000.00	1.25	8.00	150.00	3625.97
25	250000.00	1.25	8.00	200.00	1659.39
26	250000.00	1.25	8.00	100.00	2751.53
27	250000.00	2.00	2.00	150.00	2234.16
28	250000.00	1.25	5.00	150.00	3580.94
29	400000.00	1.25	5.00	200.00	1921.96

**Table 6:** Analysis of variance for the response surface modified quadratic model

**Source**	**Sum of**		**Mean**	**F**	***P*** **-value**	
	**Squares**	**df**	**Square**	**Value**	**Prob > F**	
Model	2.888×107	12	2.407×106	199.72	< 0.0001	significant
A-initial cholecalciferol concentration	4.374×106	1	4.374×106	363.01	< 0.0001	
B-tryptone	5.709×106	1	5.709×106	473.77	< 0.0001	
C-sucrose	2.534×106	1	2.534×106	210.29	< 0.0001	
D-shaking speed	7.287×105	1	7.287×105	60.47	< 0.0001	
AB	5.348×105	1	5.348×105	44.39	< 0.0001	
AC	2.555×105	1	2.555×105	21.20	0.0003	
BC	3.015×105	1	3.015×105	25.02	0.0001	
CD	4.583×105	1	4.583×105	38.04	< 0.0001	
A2	3.519×106	1	3.519×106	292.08	< 0.0001	
B2	3.698×106	1	3.698×106	306.91	< 0.0001	
C2	2.684×106	1	2.684×106	222.75	< 0.0001	
D2	1.054×107	1	1.054×107	874.35	< 0.0001	
Residual	1.928×105	16	12049.74			
Lack of Fit	1.633×105	12	13606.37	1.84	0.2921	not significant
Pure Error	29519.28	4	7379.82			
Cor. Total	2.907×107	28				
R2	0.9934					
R2adj	0.9884					
C.V.%	4.87					
Adeq precision	42.630					

**Table 7:** Validity of the model in optimal conditions

**Response**	**Software prediction Value (IU/g)**	**Validity experiment value (IU/g)**	**95% CI low**	**95% CI high**
Cholecalciferol per dry cell weight	4332.105	4428.11	4055.44	4608.77

**Figure 1:**
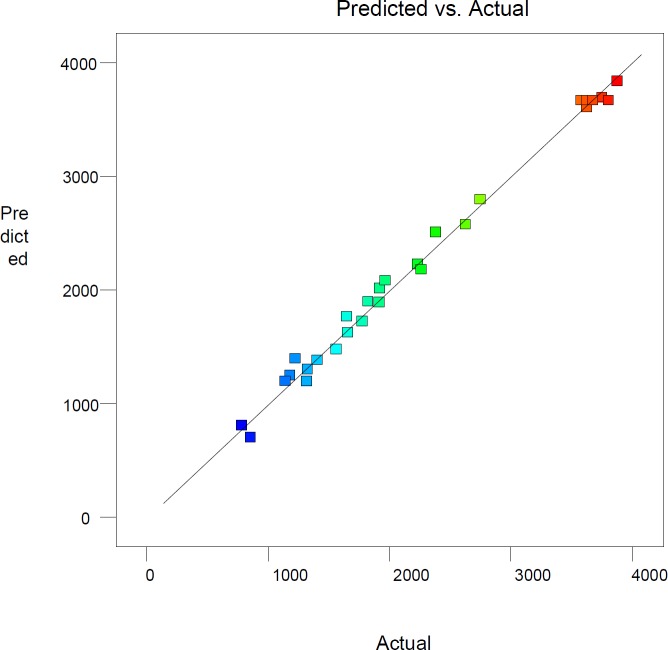
Plot of experimental versus predicted values of cholecalciferol per dry cell weight of *S. cerevisiae*

*.*

**Figure 2:**
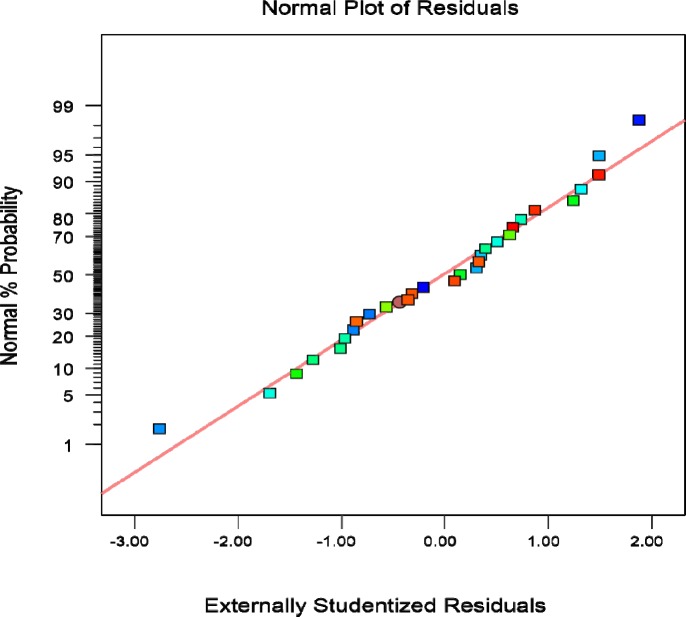
Plot of studentized residual versus predicted values of cholecalciferol amount per dry cell weight of *S. cerevisia*

**Figure 3:**
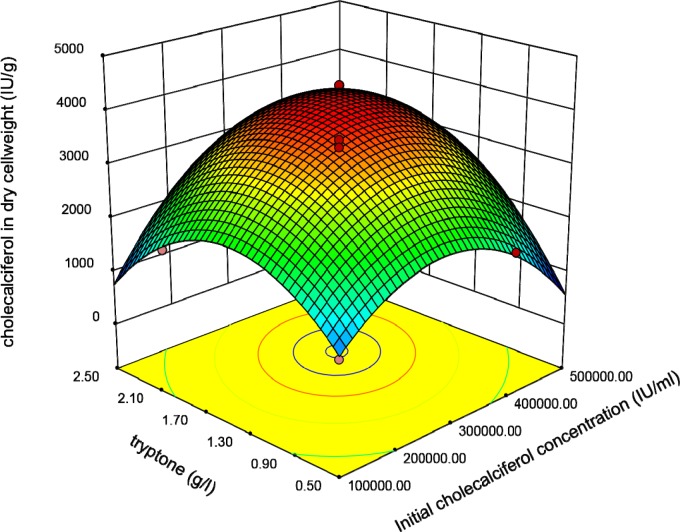
Response surface plot indicating the effect of Tryptone and initial cholecalciferol concentrations interaction on cholecalciferol amount per dry cell weight of *S.* *cerevisia*

**Figure 4:**
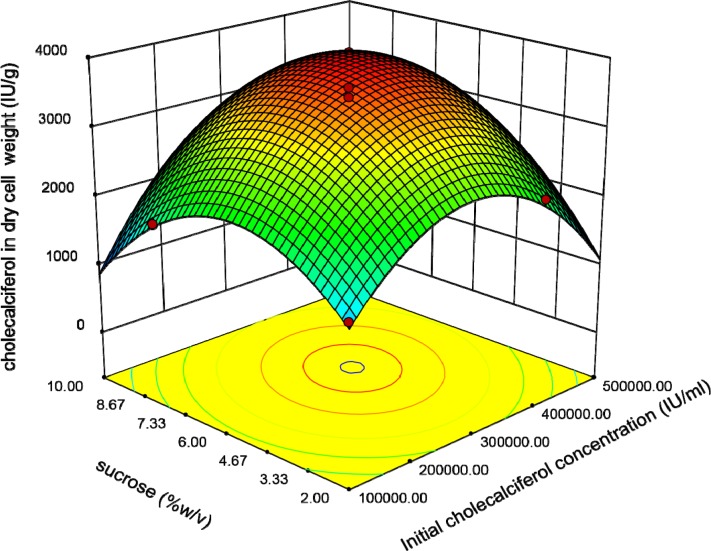
Response surface plot indicating the effect of sucrose and initial cholecalciferol concentrations interaction on cholecalciferol amount per dry cell weight of *S. cerevisia*

**Figure 5:**
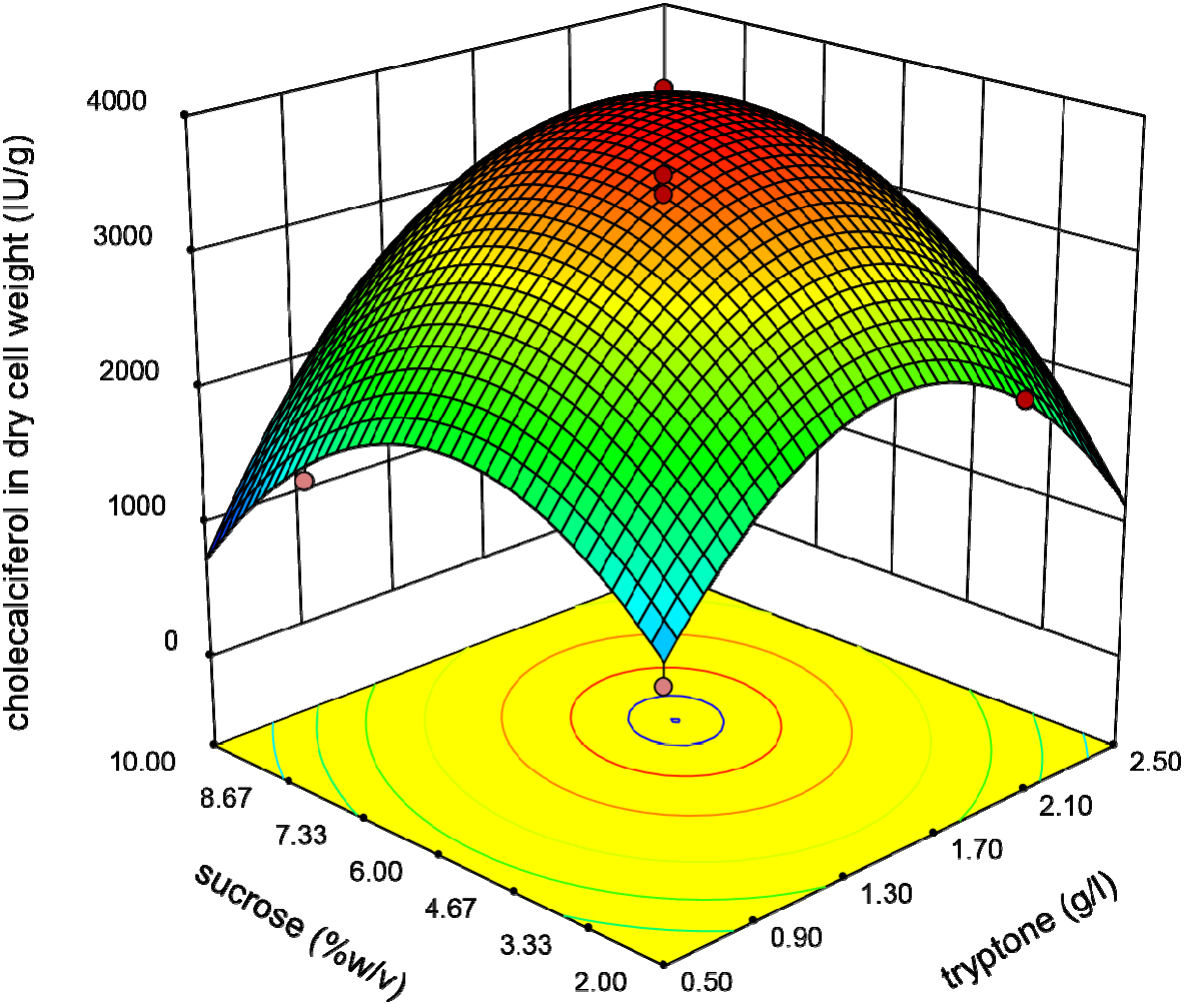
Response surface plot indicating the effect of sucrose and Tryptone interaction on cholecalciferol amount per dry cell weight of *S. cerevisia*

**Figure 6:**
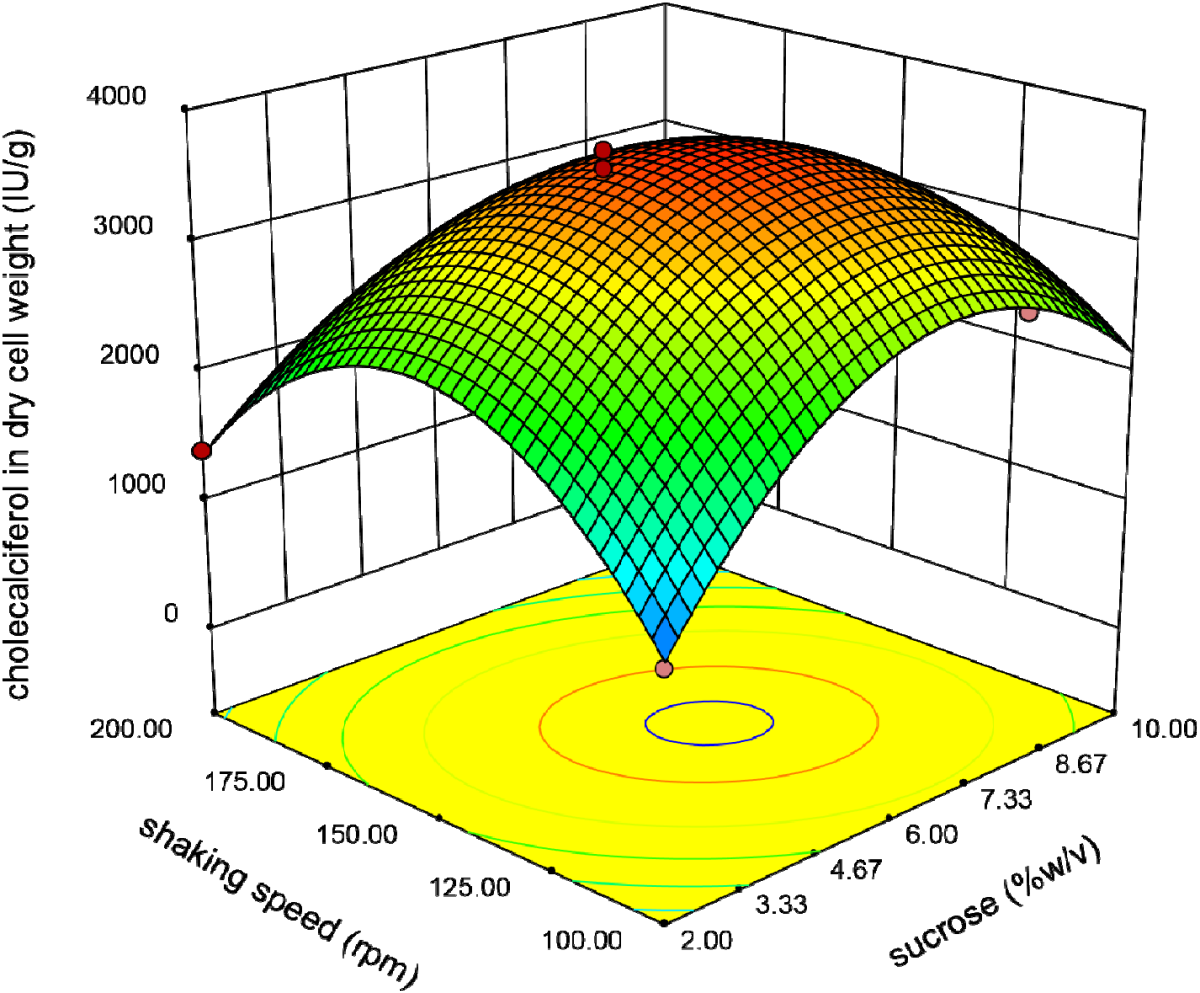
Response surface plot indicating the effect of shaking speed and sucrose interaction on cholecalciferol amount per dry cell weight of *S. cerevisia*

 Calciferol exists in several forms. Vitamin D_2 _(ergocalciferol) and vitamin D_3 _(cholecalciferol) are two main forms in human derived from ergosterol and 7-dehydrocholesterol, respectively (14). In a recent study, *S. cerevisiae *was treated with UV-B irradiation to induce alteration of yeast sterol to ergocalciferol for using as food supplement (15). While, some new investigations focused on ergocalciferol disadvantages for human and animals (16-19). Hohmann *et al.* indicated that the rats fed with ergocalciferol-enriched yeasts had lower levels of 25-hydroxy vitamin D in comparison with the rats fed with cholecalciferol (20). Nieman et al. showed that ergocalciferol supplementation reinforced abnormal exercise-induced muscle damage in some athletes (16). Tripkovic *et al.* showed that supplementation with cholecalciferol was more effective in increasing serum 25-hydroxy calciferol concentrations than ergocalciferol (17). Therefore, it seems that cholecalciferol supplementation has more beneficial effects for human health than ergocalciferol supplementation. 

Yeast *S. cerevisiae* has the ability to uptake and accumulate some micronutrients from enriched liquid media by physicochemical or metabolism-dependent reactions (21). There are some studies which have used *S. cerevisiae* as a transporter for some essential trace metals which resulted in increased bioavailability and safety of micronutrients such as iron, copper, zinc, and manganese (1, 22-24). Based on previous findings, we hypothesized that combination of yeast and cholecalciferol probably have several advantages including designing an enriched probiotic supplement, increasing half-life of vitamin by protecting against destructor agents in digestive system and sustained release of vitamin into the digestive tract for better gastrointestinal absorption. In this study* S. cerevisiae* was employed as a cholecalciferol accumulator for the first time. By using Plackett-Burman screening design, four parameters were selected for assessment of optimal conditions for enrichment of *S. cerevisiae* biomass with cholecalciferol and were optimized by using response surface methodology.

## Experimental

*Microorganism, culture condition and chemicals*

The yeast* S. cerevisiae *ATCC 9763 was maintained on Sabouraud Dextrose agar (SDA, Merck Co. Darmstadt, Germany) and stored at 4 °C. The cholecalciferol used for enrichment of *S. cerevisiae *was purchased from DSM Co. Basel, Switzerland. The other chemicals were purchased from Merck Co. Darmstadt, Germany.

*Fermentation conditions*

Sabouraud dextrose broth (SDB) medium in shake flasks was employed for optimization of culture condition. The SDB composition, that was purchased from Merck Co., (g/L) consisted of peptone from meat, 5.0; peptone from casein, 5.0; and glucose, 20.0. A 24-h culture of *S. cerevisiae* grown in SDB medium at 30 °C was used as pre-culture. The 500-ml Erlenmeyer flasks containing 200 ml of SDB were inoculated with 5% (v/v) of pre-culture to reach to an initial yeast cell concentration of about 0.1 g/L. Vitamin D_3_ (cholecalciferol) was added separately to the culture media at initial concentrations ranging from 100000-400000 IU/mL. The flasks were maintained in a shaker incubator (J Labtech, Daihan Labtech Co.) at 100-200 rpm in dark condition. The samples were taken at regular time intervals and tested for dry cell weight (g/L), and cholecalciferol uptake (IU/g).

*Dry cell weight determination*

For determination of dry cell weight, the yeast cells were isolated from SDB culture media by centrifugation (4000 ×g, 10 min) and washed twice with deionized water. The pellets were dried at 50°C under vacuum and weighed for measurement of dry cell weight.

*Measurement of cholecalciferol uptake*

*Extraction of cholecalciferol*

Extraction of cholecalciferol was carried out according to the modified method of Bligh and Dyer (25). The SDB culture medium was centrifuged for 10 min at 4000×g and the pellets were washed twice with PBS. Then, about 0.5 g of the pellets was added to 30 mL of methanol. The suspension was maintained at 25 °C and dark condition for 16 h and was sonicated in an ultrasonic bath (Q Sonica, Q700) for 20 min. The methanol was evaporated at 45 °C under nitrogen steam. Then, 10 mL of n-hexan was added to the residue and sonicated again for 15 min. The solvent was evaporated and the residue was dissolved in 1 mL of methanol and filtered before HPLC analysis.

*HPLC conditions*

HPLC was carried out with a Waters (US) 2690 system, a tunable absorbance UV-detector 486 (Waters, US) with the range of 190–800 nm, an E-600 binary pump and an auto sampler. Analyses were carried out with Millennium Software (Waters Tech.). Stock solution of standard cholecalciferol (1 mg/mL) was prepared in methanol, filtered through a 0.45 μm filter, and stored at -20 ˚C. The internal standard solution was prepared by diluting the stock solution with the mobile phase.

Cholecalciferol was separated on a Machery Nagel C18 column, 25 cm, 4.6 mm, and 5 μm. The column temperature ranged from 30 to 45 °C. Isocratic elution of mobile phase consisted of 60% methanol, 28% iso-propanol, and 12% deionized water with a flow-rate of 1 mL/min. Total run time was 22 min and the intended peak areas were integrated at 265 nm. The amount of cholecalciferol uptake was expressed as international unit (IU) per gram of dry *S. cerevisiae* cells.

*Design of experiments*

Optimization of culture conditions for enrichment of *S. cerevisiae* with cholecalciferol were carried out in three steps. In the first step, the variables were selected from previous studies. One variable-at-a-time trials were accomplished to find the range of quantitative factors and selection of appropriate qualitative parameters before screening studies. In the second step, the significant parameters were determined by Plackett-Burman screening design. Finally, in the third step, optimal levels of these parameters were evaluated by Box-Behnken technique. 

*Screening Study*

Selection of the most principal parameters among the several factors was performed by Plackett-Burman design (PBD) (26). By this technique, *n* factors in *n* + 1 experiment could be studied, where *n* is a multiple of four. Because of orthogonality in PBD, the parameters can be evaluated alone without considering their interactions.

In Table 1, eight parameters employed in the screening by PBD including temperature, shaking speed, initial concentration of cholecalciferol, inoculation volume, initial pH, Tryptone, sucrose, and dextrose (the concentration which has been added to the SDB medium) concentrations are illustrated. The codes +1 (high level) and-1 (low level) show the two different levels of the independent parameters. In PBD, the interactions between variables were omitted; therefore, a first order equation explains the model: 

Y= α_0_ +Ʃ α_ i _*x*_i_ (*i*=*1,…, k*)

where Y is the cholecalciferol uptake in yeast biomass (response), α_0_ is a constant value and α_ i_ is the regression slope.

Table 2 represents the Plackett-Burman matrix with 8 factors in 12 experiments. The symbols, (+) and (‒) show high and low levels of variables, respectively. This matrix designed by the statistical software Design-Expert^®^ version 10.0.1, Stat-Ease, Inc., Minneapolis, USA. In addition to the eight main parameters, there are three dummy factors in this matrix. Existence of dummy variables up to one-third of all parameters is helpful to estimate the random trial errors (27).

*Optimization Design*

The main response of this study, "cholecalciferol uptake in dry cell weight of *S. cerevisiae*", was optimized by response surface methodology (28). The optimal levels of variables whose effects were found significant in the screening studies, were investigated by Box-Behnken experimental design (29) using Design-Expert^®^ software version 10.0.1, Stat-Ease, Inc., Minneapolis, USA. In Table 3, the significant variables including initial cholecalciferol concentration (A), concentration of Tryptone (B), concentration of sucrose (C) and shaking speed (D) were defined at three levels of low, basal, and high which indicated ‒1, 0 and +1, respectively. For investigation of four mentioned variables, the Box-Behnken design with a set of twenty-nine trials with five central point replicates was performed. The central point trials were conducted for determination of pure error. 

A modified quadratic polynomial equation that is shown below, was the most appropriate model for description of statistical behaviors of this system: 

*Y=α0+∑αiXi+∑αiiXi* ^2^ *+∑ αijXiXj+ɛ*

where Y is the expected response, *α*_0_ is a constant value, *α*_i_ and *α*_ii_ indicate linear and quadratic influences respectively, *α*_ij_ is the quadratic influence of the interactions, *X*_i_ and *X*_j_ are value of the effective variables on response and *ε* is the error of trials. In this investigation, Y is the cholecalciferol uptake in yeast biomass. 

Statistical assessments such as analysis of variance, multiple correlation coefficient (R^2^), adjusted R^2^, Adequate precision and relative standard deviation (C.V.%) were assayed by Design-Expert^®^ software version 10.0.1 Stat-Ease, Inc., Minneapolis, USA. The significance of the modified quadratic polynomial model was determined by P value test. In this investigation, response surface plot represented the influence of parameters and their interactions on principal response.

Plot of studentized residual versus predicted response and plot of trial versus predicted response values were analyzed.

## Results and Discussion

*Plackett-Burman design (PBD) assessment*

Primary studies were accomplished to find the range of quantitative factors and selection of appropriate qualitative parameters (data not shown). Then, PBD was used for selection of the most principal parameters affecting cholecalciferol uptake in *S. cerevisiae* biomass. Statistical investigations by F test illustrated that among the parameters tested by PBD, shaking speed (B), initial concentration of cholecalciferol (C), Tryptone (D), and sucrose concentrations (H) had significant effects on the main response than the other parameters (Table 4). 

These four significant parameters had positive sign in estimate coefficient. Thus, rising their values causes increasing the cholecalciferol uptake in *S. cerevisiae* biomass. 

More statistical assessments represented that differences between the means of central points and factorial experiments in PBD were not significant (*P* > 0.05). This illustrated that optimum levels for the cholecalciferol entrapment in the yeast biomass would be near the experimental ranges selected for PBD and there was no need for steepest ascend methods.

The R^2 ^parameter of this design is approximately 98.14%, which indicates that the model is corresponding with 98.14% of the actual responses. Adjusted R^2^ is about 95.05%. The inconsistency between predicted R^2 ^and adjusted R^2 ^complains that the first order equation is not efficient for model description. 

*Box-Behnken design and trial responses*

In Box-Behnken design, trial responses, quadratic effect of each parameter and their interactions were estimated statistically. By using this experimental design, each variable was changed at low, basal and high levels when the others were kept stable. The Box-Behnken design and results of experiments are indicated in Table 5.

*Response surface methodology (RSM) analysis*

Results gained from the design of experiments revealed that the range of cholecalciferol entrapment in yeast biomass was about 785.33-3380.96 IU/g. Analysis of variance (ANOVA) of the results was done to pick out the appropriate model that fit the responses. The most relevant model has a modified second-order (quadratic) polynomial equation. As shown in Table 6, the model was significant which was approved by calculating *P*-value (*P* < 0.0001). The model acceptability was shown with lack of fit F-value of 0.292 which proposed that the lack of fit is not significant. 

The R^2 ^coefficient of this model is 0.993, which indicates that the model is corresponding with about 99% of the actual responses. Adjusted R^2^ coefficient is about 0.988. These parameters illustrate that the noise of the system is less than one percent. The predicted R^2^ of 0.975 reveals an appropriate compatibility between the value anticipated by the modified quadratic polynomial model and the trial responses. Plot of experimental versus predicted values is one of the current alternatives to assess model predictions. The straight line in this plot shows full agreement between experimental and estimated values (Figure 1).

The C.V% of 4.87 shows repeatability and integrity of the model. Adeq precision ratio of 42.630 shows an adequate signal. This parameter determines the signal to noise ratio. A ratio of more than 4 is acceptable which was seen in this model. 

Plot of studentized residual versus the value predicted by the modified quadratic model presents that deviation in the responses is acceptable and no outlier was seen in trial results (Figure 2).

The first-order, second-order and interaction coefficients of quadratic polynomial model were checked via software. Despite of interaction between initial concentration of cholecalciferol and shaking speed (AD) and interaction between Tryptone concentration and shaking speed (BD) which were insignificant (P > 0.05), other linear, squared, and interaction coefficients were significant (P < 0.05) (Table 6).

Thus, the full quadratic polynomial equation was not appropriate for model description and the modified quadratic polynomial model was employed for optimization. 

The system equation that Design-Expert software^®^ proposed is as follows:

Y = 3665.29 + 603.75 A + 689.73 B + 459.53 C – 246.42 D + 365.66 AB + 252.72 AC + 274.56 BC – 338.50 CD – 736.61 A^2^ – 755.07 B^2^ – 643.27 C^2^ – 1274.46 D^2^

where Y is the cholecalciferol uptake in dry cell weight of yeast cells. A, B, C and D are initial concentration of cholecalciferol in culture medium, concentration of Tryptone , concentration of sucrose and shaking speed, respectively. In this equation, linear coefficients of A, B, and C and interaction coefficients of AB, AC, and BC have positive sign that indicate their positive influences on the main response. Other interactions and second-order coefficients of parameters and shaking speed (D) have negative sign which could reduce the main response. All interactions except interactions between concentration of cholecalciferol and shaking speed and between Tryptone concentration and shaking speed were significant.

Interaction between initial cholecalciferol concentration and Tryptone concentration (AB) is the most significant in comparison with the other interactions. The importance of each interaction on the final response is dependent on its significance in this pattern: AB>CD>BC>AC. 

*Three-dimentional response plots*

Graphical plots especially three-dimensional response surface plots are appropriate facilities for assessment of variables. The interaction between initial concentration of cholecalciferol and Tryptone when other parameters (sucrose and shaking speed) were constant at the basal value is shown in Figure 3.

This plot reveals that the interaction coefficients of these parameters are significant. The peaked shape of the plot indicates that we could estimate the optimal level of the cholecalciferol entrapped in the yeast biomass in the range of variables. Figure 4 indicates that when the initial cholecalciferol and sucrose concentrations raised, the cholecalciferol uptake in *S. cerevisiae* biomass increased to an optimal point. The shape of plot reveals that the interaction between initial cholecalciferol and sucrose concentrations is significant too.

The relationship between Tryptone and sucrose concentrations is shown in Figure 5. It shows that raising these variables resulted in increasing of the response to an optimal level.

Finally, Figure 6 reveals the interaction between shaking speed and sucrose concentration. In all of these Figures, the shape of three-dimensional plots is convex which means positive effects of the analyzed interactions on cholecalciferol entrapment in yeast biomass. Existence of optimal levels in the range of variables confirmed the suitability of the model.

*Optimal point*

According to the results obtained from the experiments, optimum levels of the variables to maximize the main response were cholecalciferol initial concentration of 358021.16 IU/mL, Tryptone concentration of 1.82 g/l, sucrose concentration of 7.13 % (w/v) and shaking speed of 140.46 rpm. The maximum amount of cholecalciferol in dry cell weight of *S. cerevisiae* when the other variables were considered constant was anticipated to be 4332.105 IU/g. 

*Validity experiment*

To assess the validity of the optimal points obtained via the model, a trial was accomplished with the parameters proposed by the software. In this experiment, 4428.11 international unit of cholecalciferol was obtained from each gram of dried cells; which was 97.83% of the value calculated by the modified second-order model (Table 7).

The observed value is highly congruent with the software value which confirms the accuracy and reliability of the model. In this study, the most important factors that showed significant effects on the enrichment of *S. cerevisiae* with cholecalciferol were carbon source, nitrogen source, and shaking speed which are similar with the results of previous investigations about enrichment of *S. cerevisiae* by metallic elements. They reported nitrogen and carbon sources, shaking speed, temperature, and pH as the most significant variables (1, 22, 24). Although there are several studies about enrichment of yeast biomass by metallic elements, this study is the first report about entrapment of fat soluble components in the yeast cells. 

## Conclusion

This investigation is the first study that employed the probiotic yeast, *S. cerevisiae*, as an accumulator of cholecalciferol. Combination of yeast and cholecalciferol probably have several advantages including designing an enriched probiotic supplement, increasing half-life of cholecalciferol by protecting against destructor agents in digestive system and sustained release of cholecalciferol into the digestive tract for better gastrointestinal absorption. PBD studies showed that cholecalciferol absorption in yeast cells was affected by initial cholecalciferol concentration, Tryptone concentration, sucrose concentration, and shaking speed, significantly. Box-Behnken designs indicated that the reliable model which fitted to responses was a modified quadratic polynomial equation. By using RSM, cholecalciferol entrapment in yeast biomass increased from 2379.81 IU/g in SDB medium to 4428.11 IU/g in optimized medium (about 2-folds increase). 
